# Surveillance of Low-Grade Non-Muscle Invasive Bladder Tumors Using Uromonitor: SOLUSION Trial

**DOI:** 10.3390/cancers15082341

**Published:** 2023-04-17

**Authors:** Nessn Azawi, Juan Luis Vásquez, Thomas Dreyer, Cathrine Silberg Guldhammer, Rami Muthanna Saber Al-Juboori, Anna Munk Nielsen, Jørgen Bjerggaard Jensen

**Affiliations:** 1 Department of Urology, Zealand University Hospital, Sygehusvej 6, 4000 Roskilde, Denmark; 2 Institute of Clinical Medicine, University of Copenhagen, Nørregade 10, 1165 København, Denmark; 3 Department of Urology, Arhus University Hospital, 8200 Aarhus, Denmark

**Keywords:** non-muscle invasive bladder cancer, Uromonitor^®^, urinary test, surveillance, follow-up cystoscopy, low-grade recurrence

## Abstract

**Simple Summary:**

Non-muscle invasive bladder cancer (NMIBC) requires frequent cystoscopies for follow-up, which can be costly and uncomfortable for patients. This study aimed to assess the accuracy of Uromonitor, a non-invasive urinary test, in detecting NMIBC recurrence and reducing the number of cystoscopies required for surveillance. The study included 202 patients with low-grade NMIBC tumors. The results showed that Uromonitor displayed high diagnostic accuracy in detecting NMIBC recurrence, with a sensitivity of 89.7% and specificity of 96.2%. The test also had the potential to reduce the number of cystoscopies required for follow-up. These findings have important implications for NMIBC surveillance programs, as they suggest that Uromonitor could be a valuable tool for reducing patient discomfort and healthcare costs associated with cystoscopies.

**Abstract:**

Background: The surveillance of non-muscle invasive bladder cancer (NMIBC) requires frequent cystoscopies, which are costly and uncomfortable for patients. Uromonitor is a validated non-invasive urinary test for detecting NMIBC recurrence. However, data on its clinical benefit in an NMIBC surveillance program is limited. Objective: To assess the diagnostic accuracy of Uromonitor in NMIBC surveillance and its potential to limit the number of cystoscopies. Design, Setting, and Participants: The study included 202 patients with previous low-grade (LG) NMIBC tumors. Newly diagnosed patients were scheduled for flexible cystoscopy and Uromonitor test at 4, 12, and 24 months from the time of diagnosis. Patients with tumors diagnosed before entering the study underwent cystoscopy and Uromonitor test at the start of the study and 12 and 24 months from inclusion in the study. Outcome Measurements and Statistical Analysis: Sensitivity, specificity, accuracy, positive predictive value (PPV), and negative predictive value (NPV). Results and Limitations: Between February 2020 and October 2022, 202 patients were enrolled in the study. Of these patients, 171 met the eligibility criteria to perform the analysis, with a median age of 69 years, IQR (62–74), and 380 flexible cystoscopies with simultaneous Uromonitor tests. Overall, 39/171 (22.8%) patients had recurrences. Uromonitor showed a sensitivity of 89.7%, specificity of 96.2%, PPV of 72.9%, and NPV of 98.8%. In 28 cases, flexible cystoscopy was falsely positive, leading to surgery, where Uromonitor showed negative results. There were 13 cases of possible false positives for Uromonitor where flexible cystoscopy was negative. Conclusions: Uromonitor displays high diagnostic accuracy in detecting NMIBC recurrence with the potential for reducing the number of flexible cystoscopies in the follow-up of low- and intermediate-risk NMIBC. Patient Summary: We followed up on newly and previously diagnosed patients with LG NMIBC. We concluded that Uromonitor could potentially reduce the number of cystoscopies in NMIBC surveillance programs.

## 1. Introduction

Non-muscle invasive bladder cancer (NMIBC), which comprises approximately 75% of all bladder tumors, has the highest recurrence rate of all cancers, with around 70% of patients developing local recurrences despite elaborate treatment [1]. Therefore, an intensive follow-up program with surveillance cystoscopy is required and should be maintained for many years, or even throughout life, following the initial diagnosis. This surveillance program makes NMIBC the most expensive type of cancer to follow-up during a patient’s lifetime as well as by the per-patient cost [2,3]. Another drawback of the current follow-up strategy is that, due to its invasive nature, frequent cystoscopic surveillance might be perceived as highly uncomfortable and anxiety-inducing, as well as carrying a risk of infection [4,5]. The only currently used standard biomarker is urine cytology. However, this is characterized by low sensitivity, especially in patients with low-grade (LG) disease [6,7]. A high unmet need exists for cost-effective, accurate tools that successfully reduce the need for invasive cystoscopies.

Vinagre et al. reported somatic mutations in the telomerase reverse transcriptase (TERT) gene promoter in different types of cancer [8]. In bladder cancer, these somatic changes show a prevalence of up to 85% and, along with FGFR3 mutations at codons 248 and 249 [9], represent the most robust biomarkers for bladder cancer to date [10]. Based on these findings, an ultrasensitive assay (termed Uromonitor^®^) has been developed and patented to detect trace amounts of these alterations in urine samples.

Uromonitor is a non-invasive urine-based in-vitro diagnostic (IVD) test using a real-time polymerase chain reaction (PCR) with a patented combination of specific probes in defined amounts, calibrated against clinical data to deliver a test with high sensitivity and specificity. Studies have shown that Uromonitor can detect NMIBC with 100% sensitivity and 96.3% specificity regardless of tumor stage and grade (unlike cytology), with a lower false positive rate (2.3%) than that of cystoscopy (3.5%) [11].

We aimed to assess the diagnostic accuracy of Uromonitor in detecting NMIBC recurrence and to determine its potential to reduce the number of cystoscopies required for surveillance.

## 2. Materials and Methods

We conducted a multicenter, observational, prospective study. A total of 146 patients from the Urology Department of Zealand University Hospital and 25 patients from Aarhus University Hospital were included in the study. All patients with LG NMIBC diagnosed as a primary tumor or as a recurrence within the last 3 years was invited to participate in the study. Patients under active treatment with mitomycin or bacillus Calmette-Guerin (BCG) were excluded from participation. Active treatment was defined as treatment within the last 4 weeks after obtaining consent when the voided urine samples had already been taken before the flexible cystoscopy. Patients with inadequate urine quality, bacterial infection, or upper urinary tract carcinoma were excluded. The quality of urine was tested with Qubit™ 4 Fluorometer Firmware v2.19, and bacterial infection was identified by urine culture where urine test strips showed signs of infection.

Voided urine samples collected just before the flexible cystoscopy were filtered through a 0.80 μm nitrocellulose syringe filter containing a conservative storage buffer. Filters were then sent to the central laboratory at Ipatimup (Porto, Portugal). After arrival, filters were stored at 2–8 °C for a maximum of 1 month before performing the DNA extraction procedure. The results of the flexible cystoscopy and other relevant data were blinded to the Uromonitor results. The results of the Uromonitor test were labeled positive or negative according to a previously described method [12].

### 2.1. Uromonitor Analyzes

Uromonitor Kit 1—Filter Kit: Urine samples collected from patients prior to flexible cystoscopy were filtered using a Uromonitor filter kit, following their consent. Samples of 10–20 mL were filtered within an hour of collection and refrigerated at 4 °C. A pre-treated 0.80 μm nitrocellulose syringe filter (Whatman^®^ Filter—Z612545, Merck, Germany) containing a homemade conservative storage buffer was used. Filtered samples were sent to a company in Portugal within four weeks for final analysis. The company was blinded to the flexible cystoscopy results and received samples with ID numbers, which were stored in a secure database in the Department of Urology, Zealand University Hospital.

Uromonitor Kit 2—DNA Extraction and Preparation Kit: The filtered urine samples underwent final analysis in the company in Portugal. DNA was extracted and prepared for Real-Time PCR. The filter was inverted and placed on top of a cell lysis tube. A syringe with 400 μL cell lysis buffer was attached to the filter, and the solution was filtered into the cell lysis tube. The cell lysis tube was then incubated at 60 °C for 30 min under agitation. After this, protein kinase K was added, and the mixture was incubated for another 10 min at 60 °C. Then, the normalizing solution was added and vortexed for 5 s. The lysate mixture was transferred to the spin column and centrifuged. The flow-through was discarded, and the spin column was centrifuged to dry the matrix. The washing solution was added, and the column was centrifuged. The spin column was transferred to an elution tube, and an elution buffer was added. After incubation, it was centrifuged, and the eluate containing the DNA was collected. This DNA was extracted and prepared for Real-time PCR.

Uromonitor Kit 3—Real-time PCR Kit: Uromonitor Kit 3 amplified and detected TERT, FGFR3, and KRAS hotspot mutation. Each DNA stock extracted from Uromonitor Kit 2 was mixed for 5 s, and a Nanodrop UV-Vis Spectrophotometer (ND-1000/ND-2000) with EluBuffer from Urokit2 as the blank was used to quantify DNA. The specimen DNA stock was diluted according to the rules: if the DNA stock concentration was over 25 ng/μL, it was diluted to a maximum of 25 ng DNA in 1 μL of diluted DNA. If the DNA stock concentration was under 25 ng/μL, the volume needed to obtain 25 ng of DNA was calculated up to a maximum of 2.5 μL DNA per reaction. If the concentration was under 10 ng/μL, 2.5 μL DNA was used per reaction. The recommended amount of DNA per reaction was between 25 ng and 75 ng.

Cystoscopy (white light and Narrow Band Imaging) and Uromonitor tests were performed at the time of inclusion. Newly diagnosed patients were scheduled for a Uromonitor test and flexible cystoscopy at 4, 12, and 24 months. Previously diagnosed patients were scheduled for a Uromonitor test and flexible cystoscopy at 0, 12, and 24 months from the start of study inclusion (Table 1). In total, 139 patients received a single dose of intravesical Mitomycin at the time of their TUR-Bs, while 32 patients did not receive it. The decision to administer or not administer the drug was based on the center responsible for the patient’s treatment. Patients with a positive Uromonitor test and no visualization of the tumor at cystoscopy were scheduled for cystoscopy as a standard follow-up without additional intervention. Recurrent tumors detected by cystoscopy were managed according to standard guidelines. Gross hematuria, except for the first 4 weeks following a cystoscopy or transurethral removal of bladder tumor (TURBT), led to an additional interim investigation with cystoscopy.

### 2.2. Statistics

Based on preliminary data, the Uromonitor test was estimated to have 74% sensitivity and 75% specificity for detecting recurrence. The 24-month recurrence rate for the target population was assumed to be 22%. At a minimum follow-up time of 24 months, the cohort was expected to produce 30 recurrences (events).

The study was powered to estimate sensitivity within a difference of 20%, 95% confidence interval, and specificity within a difference of 10%, 95% confidence interval, or with a minimum of 30 events.

The study was considered complete when at least 30 events were reported, or 160 patients had completed a minimum follow-up of 24 months.

The ethics committee (SJ-794) and data protection agency (REG-062-2021) approved the study. The study was registered at ClinicalTrials.gov, NCT03962933.

## 3. Results

**Figure 1:**
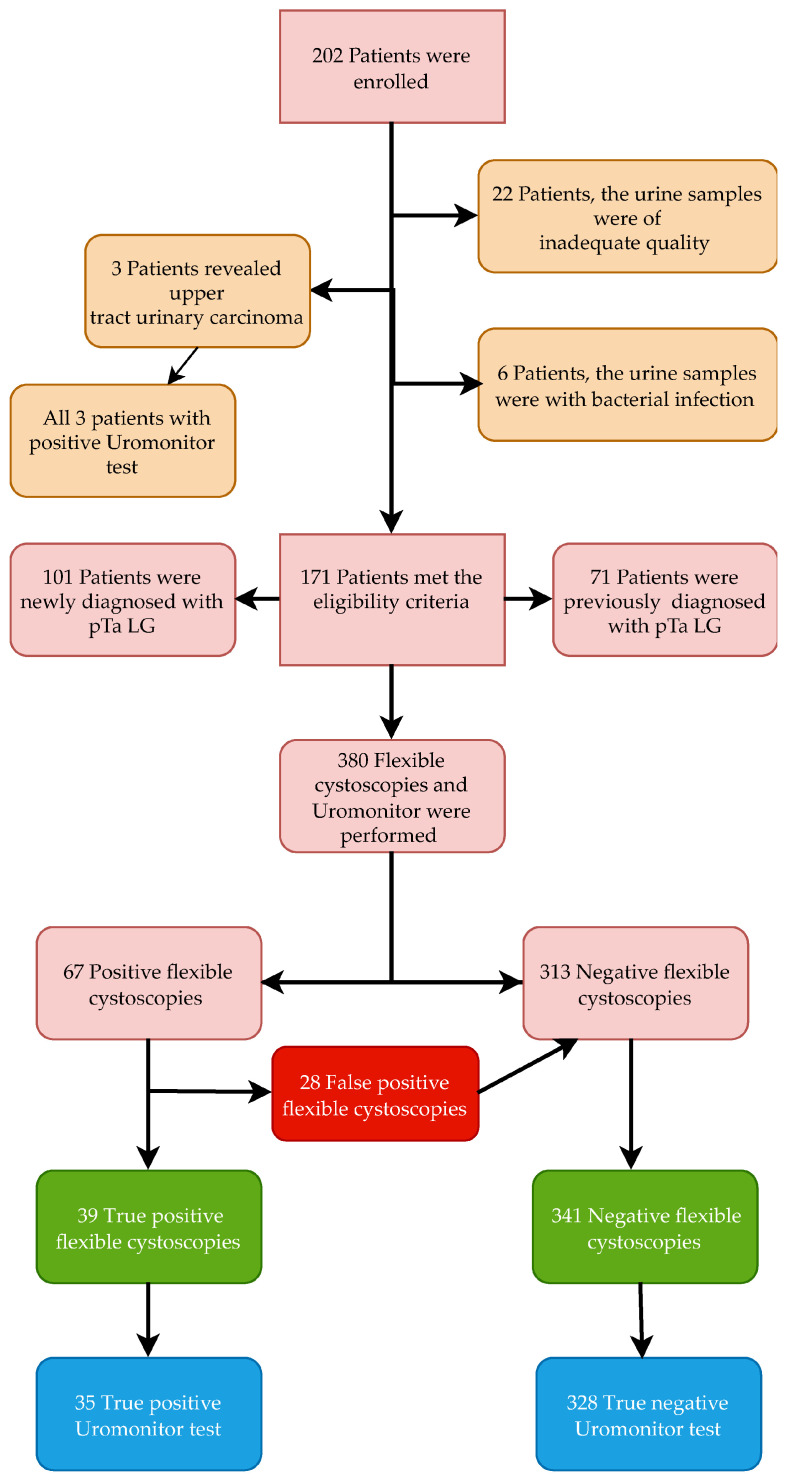
Patients inclusion process in SOLUSION trial.

Between February 2020 and October 2022, 202 patients were enrolled in the study (Figure 1). Of these patients, 171 met the eligibility criteria to perform the analysis. Thirty-one patients were excluded from the analysis. The urine samples were of inadequate quality in 22 patients, bacterial infection was revealed in six patients, and three patients revealed upper tract urinary carcinoma. All patients had previous pTa LG bladder tumors and were classified as being in the low- and intermediate-risk groups. There were no differences in patient characteristics between the low-risk and intermediate-risk groups. The median age was 69 years (IQR; 62–74) with a male-to-female ratio of 114/57; 50 patients actively smoked, 72 had smoked previously, and 49 had never smoked, see Table 2 for demographic data for 380 Uromonitor tests. There were a total of 101 patients who were newly diagnosed with pTa LG and 70 patients who were previously diagnosed with pTa LG. The median time from diagnosis to enrollment for all these patients was 10 months (IQR 6-14).

During the study period, 39/171 (22.8%) patients were diagnosed with a recurrence. The median number of tumors per recurrence was one (IQR 1–2), and the median tumor size was 2 mm (IQR 2–3 mm). No progression of disease was observed in this cohort.

Flexible cystoscopies and Uromonitor were performed 380 times during the study period.

Flexible cystoscopy revealed 67 positive findings, of which 28 were histologically negative, leaving only 39/67 (58%) true positive flexible cystoscopies.

The Uromonitor tests failed to detect bladder tumors four times. These patients had a single, small pTa LG tumor of 3–5 mm without carcinoma in situ (CIS). The Uromonitor test had a sensitivity of 89.7% (95% CI 75.8–97.1), specificity of 96.2% (95% CI 93.6–98), and accuracy of 95.5%. The negative predictive value (NPV) was 98.8% (95% CI 97–99.5). The positive predictive value (PPV) was 72.9% (95% CI 60.9–82.3), as shown in Table 3.

## 4. Discussion

Alternative surveillance strategies are required for patients with NMIBC, considering the high cost, frequency, and invasive nature of cystoscopies. Several novel urinary biomarker tests have shown promise in aiding the surveillance of bladder cancer; however, they lack the clinical validation and diagnostic accuracy to warrant clinical use. Uromonitor has been clinically validated for detecting recurrent NMIBC [11,12], and this study is the first to report the clinical benefit of Uromonitor when implemented in a standard surveillance program.

The Uromonitor device demonstrated a high sensitivity at 89.7% with a PPV of 72.9%. Two previous studies aiming to clinically validate this device have found sensitivities for recurrence detection of 73.5% and 100% with PPVs of 100% and 88.9%, respectively [11,12]. We observed 67 flexible cystoscopies with positive findings but only 39 with positive histological findings (58%). Thus, the PPV of flexible cystoscopy was not high in this study, which may be due to the varying experience of the medical staff performing the procedure as well as the quality of visualization and how systematically it was performed. Helenius et al. reported the sensitivity of flexible cystoscopy as 87% with a 98.1% PPV, comparing the true positive to computed tomography urography (CTU) scans [13], whereas Batista et al. observed a sensitivity of 79.4% for cystoscopy and a PPV of 92.1% [12].

We examined the clinical benefit of the Uromonitor test in accurately excluding a recurrence when flexible cystoscopy was falsely positive. Twenty-eight patients underwent transurethral resection of the bladder (TUR-B) due to positive flexible cystoscopies, but they were histologically negative. The Uromonitor tests were negative for all these 28 patients. The study found that by using Uromonitor, 42% (28 out of 67) of unnecessary TUR-Bs with or without anesthesia could have been avoided.

Thirteen out of forty-eight (13%) patients with positive Uromonitor tests were negative for flexible cystoscopy (we excluded the three Uromonitor-positive patients with negative flexible cystoscopy with upper urinary tract tumor). These test results may be false positives, but we cannot exclude the possibility that these patients had a recurrence too small to observe with cystoscopy. As reported by Batista et al., Uromonitor may detect microscopic lesions requiring follow-up beyond 24 months before they can be confirmed as false positives, or it is determined whether they accurately predicted further growth [12]. These 13 patients will be followed up according to the original protocol to interpret these findings.

Uromonitor showed a high NPV of 98.8%, similar to the results reported by Sampaio et al. [11,14]. Only four out of 332 patients with negative Uromonitor results revealed a recurrence during flexible cystoscopy. The rate of false negatives for flexible cystoscopy is unknown in this study as these patients were not scheduled for a TUR-B. This indicates the appropriateness of discussing using the Uromonitor test as first-line surveillance for bladder cancer rather than flexible cystoscopy. Uromonitor analysis should exclude bladder cancer with excellent reliability to enable this.

The four patients who had a recurrence not detected by the Uromonitor tests had a single, small pTa LG tumor of 3–5 mm without CIS; thus, the risk of progression, according to the European Organization for Research and Treatment of Cancer (EORTC) bladder cancer recurrence and progression score, is 1% after 1 year and 6% after 5 years [15]. If the tumor had not been detected, the patient could be scheduled for a new flexible cystoscopy after 1 year. This undetected recurrence would probably be detected by the Uromonitor analysis at the subsequent follow-up. Thus, the chance of this tumor having any consequence for the patient is minimal.

The high specificity of 96.2% for the Uromonitor test in this study indicates that only 13 (12.6%) unnecessary flexible cystoscopies would need to be performed while avoiding 332 (87.4%) flexible cystoscopies. This result confirms the potential cost-effectiveness of using the Uromonitor test during NMIBC surveillance. This should be further validated in a cost analysis.

Flexible cystoscopy has many disadvantages that are absent from Uromonitor analysis. The discomfort, risk of infection, training required for medical staff, and cost are minimized using this non-invasive test. Compared with other non-invasive novel urinary-based tests in a recent meta-analysis, the Uromonitor demonstrated the greatest diagnostic accuracy for detecting NMIBC recurrence, with significantly higher sensitivity, specificity, PPV, and NPV than the other tests [16].

The advantage of flexible cystoscopy is that the medical staff can directly view the tumor and, with experience, obtain a reliable estimation of the size, expansion, and stage of the recurrence. Uromonitor provides objective analysis, produces rapid results, and can potentially improve patients’ quality of life by reducing the need for uncomfortable cystoscopies. Our study indicates that Uromonitor analysis can be considered in NMIBC follow-up programs while shortening follow-up intervals.

If we relied solely on flexible cystoscopy, we would need to perform 380 procedures to detect the recurrence of non-muscle invasive bladder cancer (NMIBC), despite only 67 patients showing positive results on the initial cystoscopies. Furthermore, only 39 of those 67 patients were confirmed to have a true recurrence based on histological findings. On the other hand, relying exclusively on Uromonitor testing would also require 380 tests, with 48 patients requiring confirmation of the presence of tumors through flexible cystoscopy. Among these patients, thirty-five were confirmed to have true positive results based on histology, but the Uromonitor test may have missed four out of the three-hundred and eighty patients with small bladder tumors. Additionally, the negative results of flexible cystoscopy may not always accurately rule out the presence of NMIBC, with a significant proportion of cases being falsely negative. Using the Uromonitor test as a complementary diagnostic tool, the number of flexible cystoscopies required to confirm the presence of tumors could potentially be reduced to 48 out of 380 (12.6%).

The limitations of the study were its restriction to patients with pTaLG, the absence of a central pathology review, and the lack of information about cytology.

## 5. Conclusions

In the current trial, we found that the Uromonitor test is a promising tool for the surveillance of patients with pTaLG bladder tumors. A longer follow-up is required to understand the false positive rate of Uromonitor for late recurrences.

## Figures and Tables

**Table 1:** SOLUSION Trials design.

Patients Type	Inclusion	Follow-Up	Follow-Up
Newly diagnosed patients	4 months (Standard visit)	12 months (Standard visit)	24 months (Standard visit)
	Flexible cystoscopy and Uromonitor	Flexible cystoscopy and Uromonitor	Flexible cystoscopy and Uromonitor
Previously diagnosed patients (within 3 years)	0 months (Standard visit)	12 months (Standard visit)	24 months (Standard visit)
	Flexible cystoscopy and Uromonitor	Flexible cystoscopy and Uromonitor	Flexible cystoscopy and Uromonitor

**Table 2:** The demographic data of 380 Uromonitor tests.

	Negative Uromonitor Tests	Positive Uromonitor Tests
Age (median)	69 (IQR; 63–74)	67 (IQR; 57–73)
Tumor size, mm (mean)	3.5 (SD 2.8)	4.7 (SD 3.7)
Gender		
Male (N)	205	37
Female (N)	127	11
Hematuria (N)	46	11

SD = Standard deviation, N = Number.

**Table 3:** The distribution of the results of Uromonitor compared to flexible cystoscopy.

	Flexible Cystoscopies		Total
Uromonitor_Test	Negative	Positive	
Negative	328	4	332 (87.4%)
Positive	13	35	48 (12.6%)
Total	341 (89.7%)	39 (10.3%)	380

## Data Availability

The data presented in this study are available in this article.
